# Seaweed Extracts as an Effective Gateway in the Search for Novel Antibiofilm Agents against *Staphylococcus aureus*

**DOI:** 10.3390/plants11172285

**Published:** 2022-08-31

**Authors:** Maya Rima, Asma Chbani, Christine Roques, Fatima El Garah

**Affiliations:** 1 Laboratoire de Génie Chimique, Université de Toulouse, CNRS, INPT, UPS, 31062 Toulouse, France; 2 Laboratory of Applied Biotechnology, AZM Center for Research in Biotechnology and Its Applications, Doctoral School of Science and Technology, Lebanese University, El Mittein Street, Tripoli 1300, Lebanon; 3 Faculty of Public Health III, Lebanese University, Tripoli 1300, Lebanon; 4 Bacteriology-Hygiene Department, Centre Hospitalier Universitaire, Hôpital Purpan, 31300 Toulouse, France

**Keywords:** *Staphylococcus aureus*, seaweed extracts, anti-biofilm, anti-adhesion, hydrophobicity

## Abstract

Treatment of biofilm-associated infections has become a major challenge in biomedical and clinical fields due to the failure of conventional treatments in controlling this highly complex and tolerant structure. Therefore, the search for novel antibiofilm agents with increased efficacy as those provided by natural products, presents an urgent need. The aim of this study was to explore extracts derived from three algae (green *Ulva lactuca*, brown *Stypocaulon scoparium*, red *Pterocladiella capillacea*) for their potential antibiofilm activity against *Staphylococcus aureus*, bacterium responsible for several acute and chronic infections. Seaweed extracts were prepared by successive maceration in various solvents (cyclohexane (CH), dichloromethane (DCM), ethyl acetate (EA), and methanol (MeOH)). The ability of the different extracts to inhibit *S. aureus* biofilm formation was assessed using colony-forming unit (CFU) counts method supported by epifluorescence microscopic analysis. Effects of active extracts on the biofilm growth cycle, as well as on *S. aureus* surface hydrophobicity were evaluated. Results revealed the ability of four extracts to significantly inhibit *S. aureus* biofilm formation. These findings were supported by microscopy analyses. The gradual increase in the number of adherent bacteria when the selected extracts were added at various times (t_0_, t_2h_, t_4h_, t_6h_, and t_24h_) revealed their potential effect on the initial adhesion and proliferation stages of *S. aureus* biofilm development. Interestingly, a significant reduction in the surface hydrophobicity of *S. aureus* treated with dichloromethane (DCM) extract derived from *U. lactuca* was demonstrated. These findings present new insights into the exploration of seaweeds as a valuable source of antibiofilm agents with preventive effect by inhibiting and/or delaying biofilm formation.

## 1. Introduction

Although the huge marine biodiversity is far from being completely explored, previous studies have evidenced the richness of the marine world in organisms producing a library of bioactive secondary metabolites that arise from millions of years of natural selection and evolution [1,2]. Seaweed, benthic marine macroalgae widely distributed on rocky shores as well as at various sea depth, are part of sea’s treasure trove that have been used for centuries as sea vegetables, fertilizers and medicines [3]. In fact, algae are well known for their richness in unique bioactive compounds synthetized from the simple resources found in the marine environment as a natural response and a self-preservation way of facing the stressful environmental conditions [3,4].

In addition to environmental challenges (salinity, temperature changes, UV radiation exposure-etc.) encountered in seawater, algae are also exposed to other threats such as colonization/infection by undesirable microorganisms [3]. In this context, different studies have proven the wide spectrum of antibacterial activity of algal metabolites demonstrated against several Gram-negative and Gram-positive pathogenic bacteria which provides a promising gateway in the search for novel antimicrobial drugs [5].

It is obvious that the rapid emergence of multidrug resistant bacteria poses a global threat for human health which calls for intensive efforts in order to overcome the problem of antibiotic failure. Besides the well-known intrinsic and acquired genetic mechanisms involved in the bacterial resistance phenomenon, bacteria also exhibit an adaptive strategy that consists in the formation of a strongly structured cells assembly named “biofilm” [6]. Biofilms are microbial cells, embedded in a self-produced extracellular matrix and adhered to a biotic or abiotic surface. Due to the collective recalcitrance of this bacterial association towards antimicrobial agents as well as its ability to evade the host immune defenses, treatment of biofilms related infections is increasingly challenging [7].

The Gram-positive “superbug” *Staphylococcus aureus* is one of the common pathogenic bacteria well-known as a biofilm producer. Classified by the Infectious Diseases Society of America (IDSA) as member of “ESKAPE pathogens” group and defined by the World Health Organization (WHO) as a high priority in the search for novel therapeutic strategies, *S. aureus* receives a considerable attention [8,9]. This opportunistic bacterium is one of the principle human pathogens that is widely associated with hospital-acquired infections and responsible for several biofilms-related infections worldwide [10]. Besides its ability to colonize living tissues leading to severe infections such as osteomyelitis, endocarditis, and respiratory infections, *S. aureus* readily forms resilient biofilms on catheters and implanted medical devices surfaces [11].

Typically, bacterial biofilm formation occurs in three main steps, initiated by cell adhesion to a surface followed by bacterial aggregates proliferation leading to the establishment of a multi-layered structure of biofilm [12]. Then, to ensure the biofilm life cycle, a dispersion step proceeds [13].

The current treatments of *S. aureus* biofilm related infections are based on the ablation of the contaminated prosthetic devices or the administration of conventional antimicrobial agents at high concentration and for an extended period [14]. Thus, the exploration of new approaches to prevent and/or to treat *S. aureus* biofilm presents an area of active research. In this context, natural medicine, which has been used for centuries in healing and treatment of diseases, presents strong promises given the remarkable antibiofilm activity demonstrated for several natural products [15,16].

Therefore, the aim of the present study was to evaluate the potential *S. aureus* antibiofilm capacity of extracts derived from three Lebanese algae alga *U. lactuca* (Linnaeus) (green alga), *S. scoparium* (Linnaeus) Kützing (brown alga) and *P. capillacea* (S.G. Gmelin) Santelices & Hommersand (red alga). Some of these extracts have previously shown a promising antibiofilm activity against *Pseudomonas aeruginosa* by exhibiting various mechanisms of action [17]. Here, the potential antibiofilm effect of the different extracts was first assessed on *S. aureus* biofilm formation and development using colony-forming unit (CFU) counting method and supported by epifluorescence microscopy examination. Addition of the most active extracts at different stages of *S. aureus* biofilm development allowed us to deeper investigate their mode of action. The absence of a classical bactericidal effect was also assessed. Furthermore, the potential influence of the selected extracts on *S. aureus* surface hydrophobicity, known to be correlated to its adhesiveness, was evaluated by contact angle measurement method.

## 2. Results

### 2.1. Evaluation of the Antibiofilm Activity of Algal Extracts

#### 2.1.1. Effect of Extracts on *S. aureus* Biofilm Formation and Development (Extract Added at t_0_)

**Figure 1:**
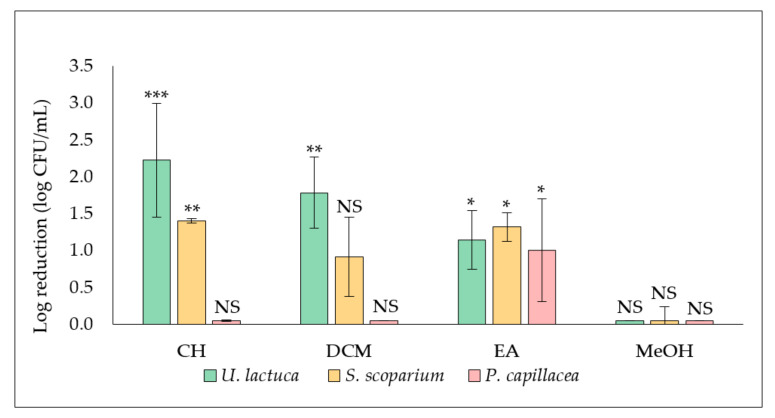
Effect of extracts (50.0 µg/mL) derived from the green alga *U. lactuca*, the brown alga *S. scoparium*, and the red alga *P. capillacea* on *S. aureus* biofilm formation and growth. Extracts were added at t_0_. Results are expressed as means of log reduction in comparison with the related untreated control (log reduction (log CFU/mL) ± SD) from three independent experiments. Statistically significant differences (***, *p*-value < 0.001, **, *p*-value < 0.01, *, *p*-value < 0.05) between log CFU/mL number in the extract treated biofilm and that in the appropriate untreated control are indicated. CH, DCM, EA, and MeOH are cyclohexane, dichloromethane, ethyl acetate, and methanol extracts, respectively. NS: not significant.

The influence of extracts (cyclohexane (CH), dichloromethane (DCM), ethyl acetate (EA) and methanol (MeOH) extracts) derived from the three tested seaweed on *S. aureus* biofilm formation and proliferation was evaluated by adding the extract at a final concentration of 50.0 µg/mL at the same time as the inoculum (t_0_) with quantification of the adhered cells after 24 h of incubation (Figure 1).

Results showed that the antibiofilm activity of *U. lactuca* extracts was the most promising. A significant reduction (***, *p*-value < 0.001) of adhered cells number was recorded in the biofilm treated with the CH extract (2.9 ± 0.7 log CFU/mL), compared to the corresponding untreated control (5.3 ± 0.4 log CFU/mL) for giving a log reduction of 2.2 ± 0.7 log CFU/mL. A significant decrease in biofilm was also observed after treatment with the DCM (**, *p*-value < 0.01) and the EA (*, *p*-value < 0.05) extracts, leading to 1.8 ± 0.5 and 1.1 ± 0.4 of log reduction (log CFU/mL), respectively.

Concerning extracts derived from the brown alga *S. scoparium*, both CH and EA extracts showed a significant effect (**, *p*-value < 0.01) by reducing 1.4 ± 0.0 and 1.3 ± 0.2 log of adherent CFU/mL, respectively. Concerning the red alga *P. capillacea*, only the EA extract showed a significant (*, *p*-value < 0.05) anti-biofilm effect (log reduction of 1.0 ± 0.7 log CFU/mL). No antibiofilm effect was recorded for all methanolic extracts as well as for CH extract derived from the red alga and DCM extracts derived from both the brown and the red alga.

**Figure 2:**
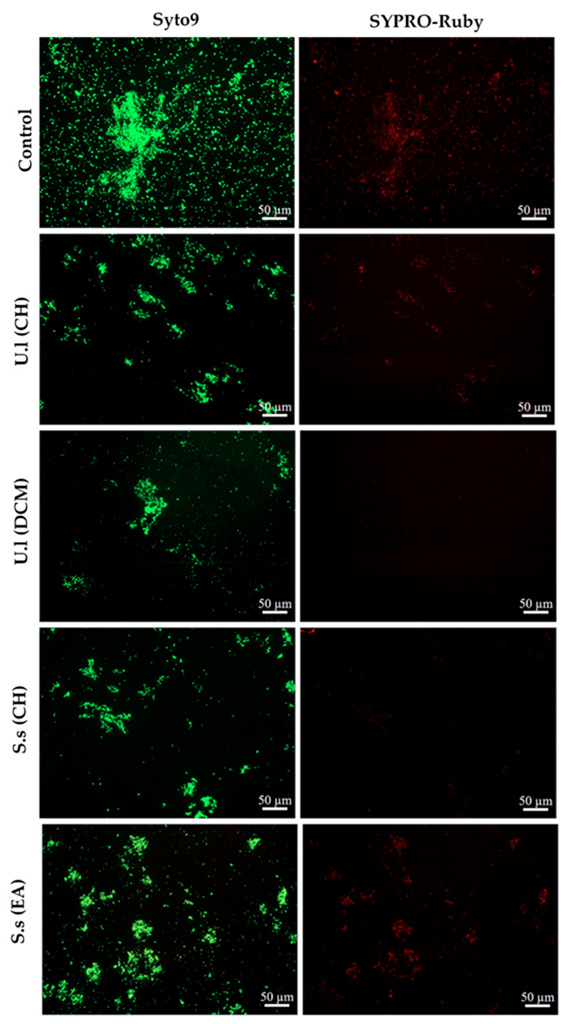
Epifluorescence microscopy images of *S. aureus* biofilms incubated in BB medium at 37 °C for 24 h without extract (control) or with selected extracts (CH and DCM extracts of *U. lactuca* and CH and EA extracts derived from *S.* *scoparium*) at 50.0 µg/mL. Biofilms were stained with Syto9 for cells visualization (green-fluorescent) and with SYPRO-Ruby for the visualization of matrix proteins (red-fluorescent). U.l and S.s are *U. lactuca* and *S. scoparium* algae, respectively. (Magnification × 20).

On the other hand, biofilms formed in presence of the most active extracts (CH and DCM extracts derived from *U. lactuca* and CH and EA derived from *S. scoparium*) were visualized by epifluorescence microscopy (Figure 2). The captured images confirmed the impact of extracts on the number of adhered cells (Syto9 staining) since the treated biofilms density was reduced compared to the untreated control. In addition, a potential effect on the protein matrix (SYPRO-Ruby staining) was recorded for DCM and CH extracts derived from the green and the brown alga, respectively.

#### 2.1.2. Determination of Biofilm Development Stage Targeted by the Selected Extracts

**Figure 3:**
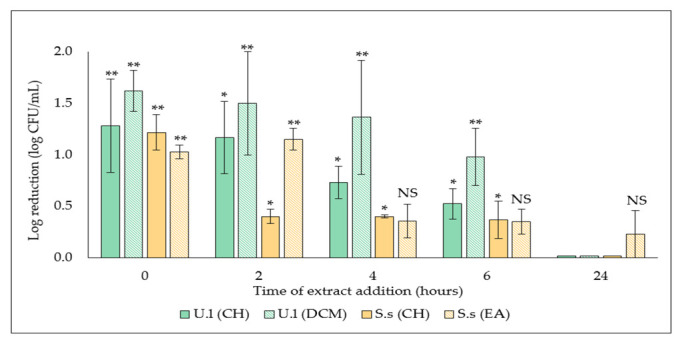
Effect of selected algal extracts (50.0 µg/mL) on *S. aureus* biofilm development phases. Extracts were added at different time points (t_0_, t_2h_, t_4h_, t_6h,_ and t_24h_). Results are expressed as means of the adhered cells number (log CFU/mL) ± SD from three independent experiments. Statistically significant differences (**, *p*-value < 0.01, *, *p*-value < 0.05) between log CFU/mL number with extract treated biofilm and that in the appropriate untreated control are indicated. NS: not significant.

With the aim of investigating the specific stage of biofilm formation affected by active extracts selected above, *S. aureus* biofilm was treated at different time points, followed by a quantification of the adhered cells after overnight incubation. Results showed the efficacy of the two extracts decreased by delaying its addition, with a total loss of efficacy on 24 h-preformed biofilm, which suggests an action on the early stages of biofilm formation (Figure 3). 

However, a significant antibiofilm activity (**, *p*-value < 0.001) of the DCM extract derived from the green alga was maintained, both when added at t_0_, with a 1.6 ± 0.2 log CFU/mL log reduction (3.9 ± 0.6 log CFU/mL versus 5.5 ± 0.8 log CFU/mL in the related untreated control) and even when added on a 6h-preformed biofilm, with a log reduction of 1.0 ± 0.3 log CFU/mL (4.9 ± 0.5 log CFU/mL versus 5.9 ± 0.6 log CFU/mL for the control).

### 2.2. Checking the Potential Bactericidal Effect of the Selected Extracts

In order to exclude the potential bactericidal effect of the selected active extracts (CH and DCM extracts derived from the green alga *U. lactuca* and CH and EA extracts derived from the brown alga *S. scoparium*) at the tested concentration (50.0 µg/mL), their effect on *S. aureus* planktonic cells was assessed. Results proved the absence of a significant effect of the selected extracts on *S. aureus* planktonic population when compared to the untreated control (Table 1) which confirms the fact that the demonstrated activity is genuinely an antibiofilm effect.

### 2.3. Effect of the Selected Extracts on S. aureus Hydrophobicity—Contact Angle Measurement Method

The potential impact of the selected extracts on *S. aureus* surface hydrophobicity was evaluated by measuring the contact angle of a drop of water deposited on a layer of previously treated bacterial cells (Table 2). The DCM extract derived from the green alga *U. lactuca* was the most potent in reducing bacterial surface hydrophobicity (***, *p*-value < 0.001) (ϴ° = 57.9 ± 8.1° versus 94.2 ± 3.8° for the untreated control). A significant effect (**, *p*-value < 0.01) of CH extract derived from the same alga was also observed (ϴ° = 85.6 ± 0.9°).

## 3. Discussion

Biofilm formation is one of the strategies adopted by bacteria to overcome treatment by antimicrobial agents as well as to escape from host immune defenses [7]. Indeed, besides the protection provided by the extracellular matrix against the penetration of antimicrobial agents, the heterogeneity within the biofilm represented by nutrient and oxygen gradients, leads to cells with different metabolic states, which promotes the resilience of this bacterial community [18,19]. Therefore, a great interest has been dedicated to the search for novel antibiofilm agents in an attempt to prevent and/or treat biofilm-related infections [20,21]. In this context, natural products are considered very promising since they have been shown to possess remarkable antibiofilm activities, with various mechanisms of action [15,22].

Interestingly, several studies have highlighted the ability of compounds isolated from marine seaweed and sponges to present a valuable input in the search for new antibiofilm agents [23,24,25]. Indeed, by living in the stressful conditions of the marine environment, many of these organisms possess sophisticated defense mechanisms that involve the natural synthesis of secondary metabolites to overcome undesirable attacks (infection, predation, biofouling…) [4].

In this context, the aim of the present study was to explore the potential ability of extracts derived from three algae, namely the green *U. lactuca*, the brown *S. scoparium*, and the red *P. capillacea* seaweed, to control biofilms formed by *S. aureus*, a common pathogen involved in hospital-acquired infections [14]. Concerning the green alga *U. lactuca*, various studies have highlighted the significant bioactivity (antimicrobial, cytotoxic, antioxidant, insecticidal activities-etc.) of its extracts (acetonic, methanolic, aqueous-etc.) [26,27,28]. Although less studied, extracts of the brown alga *S. scoparium* showed interesting biological proprieties such as antioxidant, antibacterial, anti-inflammatory, as well as cytotoxic activities [29,30]. Regarding the red alga *P. capillacea*, Ismail et al., and Shobier et al., have demonstrated the ability of its extracts to exhibit antioxidant, antidiabetic, and antifungal activities, respectively [31,32].

After preparation of the different extracts by successive maceration in four solvents (cyclohexane (CH), dichloromethane (DCM), ethyl acetate (EA), and methanol (MeOH)) with increasing polarity [17], the antibacterial activity of algal extracts against *S. aureus* was evaluated. As the objective of this study was to search for an antibiofilm effect rather than a classical antibacterial activity, the potential impact of the extracts on *S. aureus* planktonic population was investigated to ensure the observed effects were genuinely due an antibiofilm activity. Results showed none of the extracts had an antibacterial effect at tested concentration (50.0 µg/mL) (Suppl. S1). This is in accordance with two previous studies conducted by Pushparaj et al., and De Alencar et al., which indicated the absence of an inhibitory effect on *S. aureus* bacterial growth of extracts derived from the green alga *U. lactuca* (acetonic, ethyl acetate, methanolic…extracts) and the red one *P. capillacea* (hexane and ethanolic extracts), respectively [33,34]. On the other hand, Dulger et al., demonstrated the capacity of methanolic extract obtained from the brown alga *S. scoparium* to inhibit the growth of *S. aureus* but at much higher concentration [35].

The evaluation of the potential ability of extracts to inhibit *S. aureus* biofilm formation was first assessed by adding extract at t_0_. Note that the culture medium used in the evaluation of the antibiofilm activity is the low-nutritive biofilm broth (BB) which, in comparison with a rich medium, promotes biofilm formation rather than planktonic growth (Suppl S2, Figure S2). Results showed the CH extract derived from *U. lactuca* is the most promising in exhibiting an antibiofilm activity (***, *p*-value < 0.001) against *S. aureus* (Figure 1). Interestingly, this extract also showed a significant antibiofilm activity against the Gram-negative *Pseudomonas aeruginosa* in our previous study [17], which suggests the richness of *U. lactuca* in bioactive compounds with a broad spectrum of action. On the other hand, the *U. lactuca* DCM extract, as well as the CH and EA extracts obtained from *S. scoparium*, also exhibited a significant antibiofilm activity (**, *p*-value < 0.01) against *S. aureus* but not on *P. aeruginosa* [17], which indicates the involvement of different antibiofilm mechanisms of action of these extracts against these two bacterial species. Analysis by epifluorescence microscopy of *S. aureus* biofilm formed in presence of these four active extracts supports their demonstrated ability to reduce the number of adhered cells, associated with a diminution of the total amount of proteins in the biofilm matrix for DCM and CH extracts (Figure 2). It should be noted that the absence of a bactericidal effect of these four selected extracts at the tested concentration (50.0 µg/mL) was verified in order to confirm that the observed effect is definitely related to an antibiofilm activity (Table 1).

To the best of our knowledge, the brown alga *S. scoparium* has never been explored for its potential antibiofilm activity before our previous study [17]. Regarding the green alga *U. lactuca*, the study conducted by Yuvaraj & Arul, is the only one which has evaluated the antibiofilm activity of this alga against *S. aureus* [36]. The methanolic extract, prepared by a single maceration, was able to significantly reduce *S. aureus* biofilm biomass using the crystal violet (CV) staining method, commonly used in the quantification of total biofilm biomass by marking both adherent cells and matrix [37]. It should be noted that this method of biofilm quantification has been widely used in the exploration of natural products such as gallic acid and ellagic acid rhamnoside for their antibiofilm activity against *S. aureus* [38,39]. However, the CV staining method was not suitable here due to the limited quantity of matrix produced by *S. aureus* when cultured in low-nutritive medium (Suppl. S3). Furthermore, possible interference between the treatment and the CV dye may exist, as pointed out by Allkja et al., which proved the CFU counting assay followed in our study is more responsive as the most suitable method to use in treatment efficacy testing [40]. 

In order to gain insight into their potential mechanism of action, the selected extracts were added at different times point (t_0_, t_2h_, t_4h_, t_6h_ and t_24h_) during the development of *S. aureus* biofilms. Results showed a gradual loss of extracts activity by delaying their addition (t_0_, t_2h_, t_4h_, and t_6h_) (Figure 3). However, regarding the number of remaining cells after treatment by the extracts, the 24 h-old biofilm was completely resistant to the extracts which suggests they target the initial adhesion and proliferation stages. In addition, extracellular matrix of the 24 h-old biofilm can limit the penetration of extracts, thus hindering their effect. A similar behavior has been reported by Xiang et al., demonstrating the ability of aloe-emodin, a natural product derived from *Rheum officinale*, to interfere with the early stages of biofilm formation by progressively reducing *S. aureus* biofilm biomass [41]. The antibiofilm activity was explained by a reduction in the production of matrix components such as proteins and polysaccharide intercellular adhesin (PIA) involved in *S. aureus* attachment [42,43]. 

Moreover, this mode of action has been previously reported for several antibiotics such as vancomycin and moxifloxacin whose efficacy has been observed only on *S. aureus* young biofilm (6 h-old biofilm) and not on mature one (24 h-old biofilm) [44]. In *S. aureus*, the initial attachment to surface is mainly mediated by hydrophobic and electrostatic interactions followed by the production of the extracellular matrix (polysaccharides such as PIA, teichoic acids, extracellular DNA, proteins…) which is highly involved in mature biofilm resilience by providing a diffusion barrier against antimicrobial agents [14]. 

On the other hand, it is recognized that the hydrophobic proprieties of bacterial surfaces are strongly involved in adhesion to biotic and abiotic surfaces, especially to medical devices made of hydrophobic materials such as silicone and stainless steel [45]. In *S. aureus*, the attachment to abiotic surfaces is often mediated by ionic and hydrophobic interactions through surface-anchored proteins such as Bap (biofilm associated protein) and autolysin, as well as by wall teichoic acid and lipoteichoic acid [46]. Indeed, the prevalence of hydrophobic patches, compared to hydrophilic ones, on the surface of *S. aureus* was demonstrated in the study conducted by Forson et al., in which the adhesion was favored on the hydrophobic surface [47]. In addition, Kouidhi et al., have highlighted a correlation between the surface hydrophobicity of various *S. aureus* strains associated with dental caries and their adhesiveness on polystyrene plates [48]. In this context, the potential effect of the selected extracts on *S. aureus* hydrophobicity was assessed in an attempt to elucidate their potential mechanism of action. For this purpose, the sessile drop technique, which consists in measuring the contact angle of a water drop on a bacterial film, was adopted. Indeed, the contact angle presents an indirect and proportional measure of the hydrophobicity as a higher contact angle indicates a greater surface hydrophobicity [49]. 

Results revealed the high hydrophobicity of *S. aureus* cells (ϴ = 94.2 ± 3.8°) (Table 2). Interestingly, a significant reduction in the hydrophobicity of *S. aureus* cells treated either with CH (**, *p*-value < 0.01) or DCM (***, *p*-value < 0.001) extracts derived from the green alga was observed. Combined with the demonstrated ability of these two extracts to reduce *S. aureus* biofilm when added at early stages of biofilm formation (up to 6 h) (Figure 3), their potential mechanism of action may be based on the inhibition of the initial adhesion by decreasing the surface hydrophobicity. This particular mechanism of action has already been described for brodimoprim, an antibacterial agent whose ability to reduce the adhesiveness of *S. aureus* to human epithelial buccal cells has been correlated with a decrease in bacterial surface hydrophobicity [49]. In addition, Allegrone et al., have demonstrated the capacity of natural rhamnolipids and Triton^TM^—X100 (a synthetic surfactant) to significantly reduce *S. aureus* surface hydrophobicity, as well as to inhibit its adhesion to a surfactant-precoated silicone surface [50]. 

Regarding the chemical composition of extracts, we previously have identified several phenolic compounds by GC-MS analysis [17], some of which are known for their broad spectrum of biological activity such as 2,4-di-tert-butylphenol [51]. To assess its potential implication in the demonstrated antibiofilm activity of the active extracts, this phenolic compound was quantified in the extracts (Suppl S4, Figure S4, Table S4). Its presence in low quantity (<25 µg/mL at which concentration 2,4-di-tert-butylphenol exhibit a significant antibiofilm activity, Suppl S5, Figure S5) in both active and inactive extracts makes us reconsider the involvement of this compound in the antibiofilm activity of the active extracts. In this context, further bio-guided fractionation will allow to isolate and identify the molecule(s) responsible for the promising antibiofilm activity of the active extracts.

## 4. Materials and Methods

### 4.1. Collection of Algal Materials and Extract Preparation 

The algae evaluated in this study are the green alga *Ulva lactuca* (Linnaeus) the brown alga *Stypocaulon scoparium* (Linnaeus) Kützing, and the red alga *Pterocladiella capillacea* (S.G. Gmelin) Santelices & Hommersand which were collected from the the Mediterranean Sea along the northern coast of Lebanon [17]. Extracts were prepared by successive maceration in solvents with increasing polarity (cyclohexane, dichloromethane, ethyl acetate, and methanol) as previously described [17]. Stock solutions were prepared by dissolving the dry crude extracts in sterile distilled water (SDW) at 100.0 µg/mL, using an ultrasonic bath (VWR ultrasonic cleaning bath, 45 KHz) for almost 6 h to promote the solubility. Stock solutions were then sterilized by filtration through a syringe filter (Cellulose Acetate Syringe Filter, 0.45 µm) purchased from Dutscher, Bernolsheim, France. 

### 4.2. Bacterial Strain and Culture Media 

The bacterial strain used in this study is *Staphylococcus aureus* (CIP 4.83), purchased from the collection of Pasteur Institute (Paris, France) and preserved at −80 °C. Before each experiment, two successive overnight subcultures were realized on Trypticase soy agar TSA (BioMérieux, Crapone, France) and incubated under aerobic conditions at 37 °C. The antibiofilm activity assays were conducted in the previously selected low-nutritive medium named biofilm broth (BB) in order to create stressful conditions and subsequently promote biofilm formation and adherent cells growth rather than planktonic growth [19]. 

The BB 10X is composed of FeSO_4_ · 7H_2_O (0.005 g/L), Na_2_HPO_4_ (12.5 g/L), KH_2_PO_4_ (5.0 g/L), (NH_4_)_2_ SO_4_ (1.0 g/L), lactose (0.25 g/L), yeast extract (1.0 g/L), vitamin assay casamino acids (1.0 g/L) and MgSO_4_ · 7H_2_O (0.2 g/L) [19]. Except for yeast extract (Bacto^TM^, ThermoFisher scientific, llkirch, France) and vitamin assay casamino acids (Difco^TM^, ThermoFisher Scientific, llkirch, France), all these compounds were purchased from Sigma-Aldrich, St. Quentin Fallavier, France. 

### 4.3. Evaluation of the Antibiofilm Activity of Extracts 

First, the ability of extracts derived from the three algae to inhibit *S. aureus* biofilm formation and development (extract added at t_0_) was determined. The biofilms formed in the presence of the potentially active extracts were visualized by epifluorescence microscopy. Then, the most active extracts were selected in order to investigate the targeted biofilm development phase by adding extract to a *S. aureus* biofilm at various development stages (t_2h_, t_4h_, t_6h_, and t_24h_). It should be noted that in all assays, the quantification of *S. aureus* biofilm was performed by counting the adhered cells recovered by scraping as previously described [52]. Assays were performed in triplicate. 

#### 4.3.1. Effect of Extracts on *S. aureus* Biofilm Formation and Development (Extract Added at t_0_)

The influence of extracts on the number of adhered cells was evaluated following the CFU counts method previously described by Khalilzadeh et al., with some modifications [52]. 

The bacterial suspension used in this assay was prepared in the low-nutritive medium BB (2X) and was adjusted to 10^8^ CFU/mL (OD_640nm_ = 0.150) followed by ten-fold serial dilution up to 10^−6^ with the same medium. Then, 1.0 mL of the 10^−6^ dilution (equivalent to 10^2^ CFU/mL) was introduced into the wells of a 24-well plates (Falcon, TC-treated, polystyrene). 1.0 mL of algal extract (100.0 µg/mL) was added at t_0_, corresponding to a final concentration of 50.0 µg/mL. Algal extract was replaced by 1.0 mL of SDW in biofilm growth control. Wells containing 1.0 mL of SDW + 1.0 mL of un-inoculated BB (2X) medium was considered as sterility control. After overnight incubation at 37 °C, wells’ content was discarded followed by rinsing (×2) with 2.0 mL of SDW in order to remove unattached planktonic cells. Adhered cells were then recovered by scraping for 1 min with a sterile spatula into 1.0 mL of SDW followed by a ten-fold serial dilution (from 10^−1^ to 10^−6^) [52].

900.0 µL of each dilution was then inoculated by inclusion in TSA agar plates. After 48 h of incubation at 37 °C, the numbers of CFU were determined by considering only plates with 15 to 300 CFU. The adhered biomass was then calculated and subjected to logarithmic transformation by the following Formula (1). The logarithmic reduction with respect to the corresponding untreated control was calculated using the formula below (2).

(1) $$\mathrm{Log}\;\mathrm{of}\;\mathrm{adhered}\;\mathrm{biomass}\;\left(\log\mathrm{CFU}/\mathrm{mL}\right)=\log\;\frac{\mathrm{number}\;\mathrm{of}\;\mathrm{colonies}\;(\mathrm{CFU})}{\mathrm{Dilution}\;\mathrm{factor}\;\times \;\mathrm{inoculated}\;\mathrm{volume}}$$

(2) $$\mathrm{Log}\;\mathrm{CFU}/\mathrm{mL}\;\mathrm{reduction}=\mathrm{logCFU}/{\mathrm{mL}}_{\mathrm{control}\;}-\;\mathrm{logCFU}/{\mathrm{mL}}_{\mathrm{treated}\;\mathrm{biofilm}}$$

#### 4.3.2. Epifluorescence Microscopic Analysis of Treated Biofilms (Extract Added at t_0_)

Biofilms formed in presence of the potentially active extracts were visualized by epifluorescence microscopy. For this analysis, *S. aureus* biofilms were grown as described above but in a 6-well microplate (Falcon, TC-treated, polystyrene) and with a total volume of 6.0 mL (3.0 mL of *S. aureus* bacterial suspension prepared in BB 2X (10^2^ CFU/mL) + 3.0 mL of tested extract or 3.0 mL of SDW for the biofilm growth control).

After 24 h of incubation at 37 °C and in order to evaluate the potential effect of extracts on *S. aureus* biofilm (cells number and matrix), 1.0 mL of SYPRO Ruby stain (Invitrogen^TM^, FilmTracer^TM^, SYPRO^TM^ Ruby biofilm matrix stain) was added after discarding wells content. This stain binds to most classes of proteins including glycoproteins, lipoproteins, phosphoproteins and fibrillar proteins. After 30 min of incubation in dark at room temperature, wells were carefully washed twice with 1.0 mL of SDW. Six mL of SDW were then added supplemented with 1.0 µL of Syto9 stain (5 mM, Invitrogen^TM^, ThermoFisher Scientific, llkirch, France) for cell visualization. 

Microscopic observations were made with Zeiss—Axiotech microscope using a 20 ×/0.50 (Zeiss, EC Plan-Neofluar) objective and equipped with an HXP 120 C light source. Images were acquired with a digital camera (Zeiss AxioCam ICm 1) and then the set of photos was processed with ZEN software.

#### 4.3.3. Determination of Biofilm Development Stage Targeted by the Selected Extracts 

Extracts that showed the most significant activity (***, *p*-value < 0.001; **, *p*-value < 0.01) (in comparison to the biofilm growth control) on *S. aureus* biofilm formation were selected. With the aim of specifying biofilm development phase targeted by these active extracts, *S. aureus* biofilm was treated at different stages of growth as outlined in Table 3.

Briefly, at t_0_, 1.0 mL of bacterial suspension (10^2^ CFU/mL) prepared in BB (2X) medium was introduced into the wells of a 24-well plate, either with 1.0 mL of SDW (control and biofilms treated at 2, 4, 6, and 24 h) or with 1.0 mL of extract (biofilm treated from t_0_) (final concentration 50.0 µg/mL). For sterility check, 1.0 mL of un-inoculated BB (2X) + 1.0 mL of SDW were introduced into control wells. Plate was then incubated at 37 °C. 

At different time points (t_2h_, t_4h_, t_6h_, t_24h_), the formed biofilm was washed twice with 2.0 mL of SDW and 1.0 mL of BB (2X) medium was added supplemented with 1.0 mL of extract (final concentration 50.0 µg/mL). The extract solution was replaced by 1.0 mL of SDW in the corresponding control wells.

Plates were incubated at 37 °C and adhered cells were recovered by scraping after 24 h or 48 h of incubation. After quantification and logarithmic transformation of the number of adhered cells, the logarithmic reduction with respect to the corresponding untreated control, was calculated using the formula above (3).

### 4.4. Checking the Potential Bactericidal Activity of the Selected Extracts

In order to exclude a potential bactericidal effect of the active extracts (CH and DCM *U. lactuca* extracts and CH and EA *S. scoparium* extracts) at the tested concentration (50.0 µg/mL), their effect on *S. aureus* planktonic cells was assessed. The protocol developed by Feuillolay et al., was used in this assay [53]. Briefly, 2.0 mL of *S. aureus* bacterial suspension (10^5^ or 10^2^ CFU/mL) prepared in BB (2X) medium and supplemented with 2.0 mL of sterile distilled water were incubated for 24 h at 37 °C. Water was replaced by 2.0 mL of extracts in the sample tubes. The potential bactericidal activity of extracts was determined for both inoculi (10^5^ and 10^2^ CFU/mL). Tubes were maintained under agitation (100 rpm) in an orbital shaker (MaxQ 4000, ThermoFisher Scientific, Waltham, USA). 

The number of planktonic cells was monitored by plating on TSA agar plates and incubation at 37 °C for 24 h. The bactericidal effect of extracts was determined by calculating ratio between the number of bacteria (log CFU/mL) present in the sample tube and those in the control tube.

### 4.5. Effect of the Selected Extract on S. aureus Hydrophobicity—Contact Angle Measurement Method 

In order to evaluate the effect of the selected extracts on *S. aureus* hydrophobicity, the sessile drop technique, which consists in measuring the contact angle of a water drop on a bacterial layer, was carried out. The protocol described by Elabed et al., was adopted with some modifications [54]. Briefly, 5.0 mL of *S. aureus* suspension prepared in BB (2X) medium (OD_640nm_ = 0.3) was added to 5.0 mL of extract (final concentration 50.0 µg/mL). The extract solution was replaced by 5.0 mL of SDW in the control tube. After 2 h of incubation at 37 °C and in order to remove extracts, bacteria were recovered by vacuum filtration on a sterile cellulose acetate membrane filter (0.45 µm, Merck-Sigma, Saint-Quentin Fallavier, France) that was dehydrated for almost 30 min at room temperature prior to the measurement of the contact angle. 

The contact angle between a water drop (1–2 µL) and the bacterial lawns was then measured under ambient conditions using a Digidrop contact angle meter (GBX Scientific Instruments, Romans-sur-Isère, France). Measurements were computed automatically by Windrop++ software. It should be noted that the measurement should be done within 3–4 s after depositing the drop, before its penetration into the bacterial layer. Contact angles were determined at 5 random points per bacterial film. Results are expressed as mean contact angle ± SD. 

### 4.6. Statistical Analysis

All values are expressed as mean ± SD for three independent experiments. The student t-test was used to calculate the significance of the differences between the mean effects of the extract and those for the associated untreated control after checking equality of variances with Levene’s test (*p*-value < 0.05). Statistically significant values were defined as a *p*-value (* < 0.05, ** < 0.01 or *** < 0.001). SPSS 22.0 software (SPSS, IBM Corporation, Armonk, NY, USA) was used in the statistical analysis. 

## 5. Conclusions

In the present study, the exploration of various extracts derived from three algae for their potential ability to present an antibiofilm activity against *S. aureus* permitted the selection of four promising extracts: the CH and DCM *U. lactuca* extracts and the CH and EA *S. scoparium* extracts. Their significant antibiofilm effect was demonstrated to target the early stages of biofilm formation. Regarding the potential antibiofilm mechanism of action exhibited by the *U. lactuca* CH and DCM extracts, a decrease in *S. aureus* surface hydrophobicity may explain their ability to hinder the bacterial adhesion and/or to delay biofilm proliferation. On the other hand, a reduction in matrix proteins was observed in biofilms formed in presence of DCM and CH extracts derived from *U. lactuca* and *S. scoparium*, respectively. In light of these encouraging results, further experiments are envisaged to decipher the possible mechanism of action of the selected active extracts, particularly through molecular analysis. Furthermore, it will be interesting to go further in the analysis of the chemical composition of the active extracts in an effort to isolate highly active molecules. Overall, the findings of this study pave the way for possible future applications of seaweeds as preventive treatment against bacterial biofilms.

## Figures and Tables

**Table 1:** Evaluation of the potential bactericidal activity of the selected extracts (final concentration: 50.0 µg/mL) on S. aureus (10^5^ CFU/mL or 10^2^ CFU/mL). The number of planktonic cells was measured after 24 h of incubation at 37 °C under agitation. Results are expressed as ratio between the number of bacteria (log CFU/mL) in sample tube and those in the control tube.

Initial Bacterial Suspension	Green Alga *U. lactuca*		Brown Alga *S. scoparium*	
	CH	DCM	CH	EA
10^5^ CFU/mL	0.98 ± 0.00	0.96 ± 0.01	0.99 ± 0.02	0.95 ± 0.00
10^2^ CFU/mL	1.00 ± 0.00	0.99 ± 0.00	0.99 ± 0.00	1.02 ± 0.00

**Table 2:** Effect of the selected extracts (50.0 µg/mL) on *S. aureus* surface hydrophobicity assessed by measuring the contact angle ϴ°. Results are expressed as mean of ϴ° determined at 5 random points per bacterial film (ϴ° ± SD). Statistically significant differences (***, *p*-value < 0.001, **, *p*-value < 0.01) between the extract treated bacterial layer and the untreated control one are indicated. ^NS^: not significant.

Sample	Contact Angle ϴ°	Water Droplet Deposited on the Bacterial Layers
Control	94.2 ± 3.8°	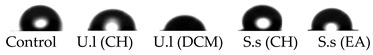
U.l (CH)	85.6 ± 0.9° **	
U.l (DCM)	**57.9 ± 8.1°** ***	
S.s (CH)	94.1 ± 4.1° ^NS^	
S.s (EA)	90.8 ± 6.3° ^NS^	

**Table 3:** Protocol for the addition of extract at different time points.

Stage of Biofilm Formation	Time Point of Extract Addition				
	0	2 h	4 h	6 h	24 h
0	↓ +	↓	↓	↓	↓
2 h		+			
4 h			+		
6 h				+	
24 h	Scraping time				+
48 h	_				Scraping time

“↓” is inoculation time point and “+” is extract addition time point.

## Supplementary Materials

The following are available online at https://www.mdpi.com/article/10.3390/plants11172285/s1, S1: Effect of extracts on *S. aureus* planktonic growth—MIC determination; S2: Comparison between *S. aureus* planktonic growth kinetics in the rich medium MHB and the low-nutritive biofilm broth BB; Figure S2: Planktonic growth kinetics of *S. aureus* in MHB and BB medium; S3: Quantification of *S. aureus* biofilm by crystal violet staining method; S4: Quantitative analysis of 2,4-di-tert-butylphenol in extracts—GC/MS analysis; Figure S4: The calibration curve of 2,4-di-tert-butylphenol indicating the peak areas versus concentrations. Table S4: Concentrations of 2,4-di-tert-butylphenol determined in extracts. S5: Evaluation of the antibiofilm activity of 2,4-di-tert-butylphenol against *S. aureus*; Figure S5: Effect of 2,4-di-tert-butylphenol (50.00, 25.00, 12.50, and 6.25 µg/mL) on *S. aureus* biofilm formation and growth.
