# Supplementary material for: Seaweed Extracts as an Effective Gateway in the Search for Novel Antibiofilm Agents against Staphylococcus aureus
Source: Plants (Basel). 2022 Aug 31;11(17):2285. doi: 10.3390/plants11172285 (PMC9459781; doi:10.3390/plants11172285)
Supplement: Supplementary file 1 [file plants-11-02285-s001.zip › plants-1853632-supplementary.pdf]

Supplementary Materials

S1. Effect of extracts on *S. aureus* planktonic growth – MIC determination

Prior to the assessment of their potential effect on biofilm, the evaluation of antibacterial activity of extracts on *S. aureus* is essential for ensuring the use of a sub-MIC concentration in antibiofilm activity assays. For this purpose, the minimal inhibitory concentration (MIC) of all extracts against *S. aureus* was determined using the broth microdilution method, according to the guidelines of CA-SFM/EUCAST 2020. Briefly, 100.0 µL of algal extract stock solution (100.0 µg/mL) were introduced into the wells of the first column of a sterile 96-well microtiter plate (Falcon, TC-treated, polystyrene) and subjected to 2-fold serial dilutions with Mueller-Hinton broth (MHB) (100.0 µL/well) to achieve final concentrations ranging from 50.0 to 0.098 µg/mL. The bacterial suspension used in this assay was prepared in SDW and adjusted to an optical density of 0.150 at 640 nm, corresponding to a concentration of 10^8 CFU/mL. This suspension was then subjected to a 2-fold dilution in SDW prior to the inoculation of the microtiter-plate using a manual multipoint inoculator (1.0 µL), in order to obtain a final concentration of 5 x 10^5 CFU/mL. Note that wells in the last column were used as sterility controls (SDW + MHB). The previous column was dedicated to growth control (SDW + MHB + inoculum). After incubation at 37˚C for 24h, the MIC, defined as the lowest concentration of the tested extract which can prevent the visible bacterial growth was determined. Assays were performed in duplicate. For all extracts, no antibacterial activity was demonstrated at the highest concentration tested (50.0 µg/mL). Therefore, the concentration adopted in the antibiofilm activity was 50.0 µg/mL.

S2. Comparison between *S. aureus* planktonic growth kinetics in the rich medium MHB and the low-nutritive biofilm broth BB

S. aureus growth kinetics curves in the rich medium MHB and the low-nutritive biofilm broth BB were performed. Briefly, 100 µL of tested media (MHB and BB 2X) were introduced into the wells of a sterile 96-well microtiter plate (Falcon, TC-treated, polystyrene) supplemented with 100 µL of sterile distilled water. *S. aureus* suspension used in this assay was prepared in sterile distilled water and adjusted to an optical density of 0.150 at 640 nm, corresponding to a concentration of 10^8 CFU/mL followed by dilution (1:10) to achieve a concentration of 10^7 CFU/mL. Then, the microtiter-plate was inoculated using a manual multipoint inoculator. Wells with 100 µL of distilled water and 100 µL of the tested medium were used as sterility controls. The plate was incubated at 37°C for 24h in a microplate reader (CLARIOstar Plus Plate reader – BMG Labtech) under continuous agitation. $OD640_{nm}$ measurement was carried out every one hour. The measured values were plotted as a function of incubation time (Figure S2).

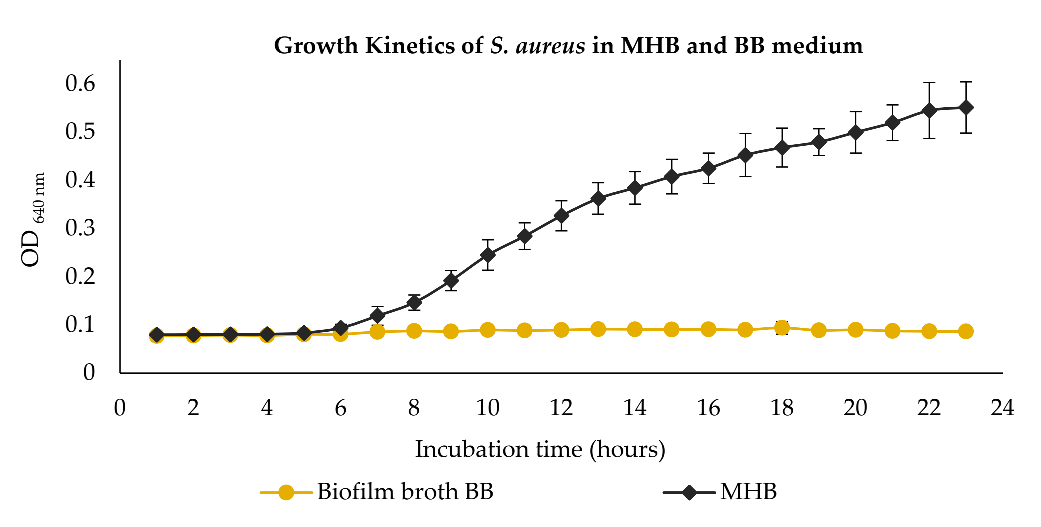

Figure S2. Planktonic growth kinetics of *S. aureus* in MHB and BB medium. Results are expressed as means ± SD of $OD_{640\ nm}$. MHB and BB are Mueller-Hinton broth and biofilm broth, respectively.

S3. Quantification of *S. aureus* biofilm by crystal violet staining method

In order to screen extracts derived from the three algae for their ability to inhibit *S. aureus* biofilm formation and growth, the crystal violet staining method, commonly used for the quantification of bacterial biofilms biomass (adhered cells + matrix), was first adopted. Briefly, after overnight incubation of the treated biofilms, they were washed twice with 2.0 mL of SDW to remove non-adherent planktonic cells. The plate was then air-dried for 1h. To stain the adhered biomass, 2.0 mL of an aqueous 1% CV solution was added to the wells and consecutively incubated for 15 min at room temperature. In order to remove the excess stain, wells were rinsed twice with 2.0 mL of SDW followed by drying for 30 min before quantification. 1.0 mL of ethanol was finally added to extract bound stain prior to the absorbance measurement (OD_{570nm}).
Results showed that the evaluation of the antibiofilm activity of extracts against *S. aureus* by this method is not feasible in our culture conditions due to the low quantity of biofilm biomass detected in the control wells (OD_{570nm} = 0.06 $\pm$ 0.03). For this reason, CFU counts method was used in this study.

S4. Quantitative analysis of 2,4-di-tert-butylphenol in extracts – GC/MS analysis

The calibration curve of 2,4-di-tert-butylphenol (97%, CAS Number: 96-76-4, Acros Organics, France) was carried out by GC-MS analysis in order to quantify this phenolic compound in extracts. For this purpose, various solutions of 2,4-di-tert-butylphenol (100.0, 50.0, 40.0, 30.0, 20.0, 10.0, and 5.0 µg/mL) were prepared in acetonitrile ($\geq$ 99.9 %, Sigma-Aldrich, France). On the other hand, extracts were prepared at a concentration of 2.5 mg/mL in the corresponding extraction solvent (cyclohexane (CH), dichloromethane (DCM), ethyl acetate (EA), or acetonitrile for methanolic (MeOH) extracts). Analyses we performed using GC-MS system (TRACETM 1310—ThermoFisher Scientific) equipped with a Rtx-502.2 fused silica capillary column (30 m in length, 0.25 mm in diameter, 1.4 µm in film thickness). The column oven temperature was programmed as follows: initial temperature was 50 ◦C (for 2 min) then gradually increased to 150 ◦C (for 5 min) at a rate of 20 ◦C/min, and finally increased to 290 ◦C at a rate of 10 ◦C/min and maintained for 10 min. Ionization of the sample components was performed in electron impact mode (EI, 70 eV) with 220 ◦C as ion temperature. The injector and detector temperatures were 250 and 220 ◦C, respectively. Hydrogen was used as carrier gas at a flow rate of 1.0 mL/min. The injection volume of the prepared solutions (2.5 mg/mL) was 5.0 µL. The total running time of the GC–MS system was 36 min. Finally, the calibration curve of 2,4-di-tert-butylphenol was plotted based on the measurement of peak area using Xcalibur software (Figure. S4). Its quantity in extracts was then determined (Table. S4).

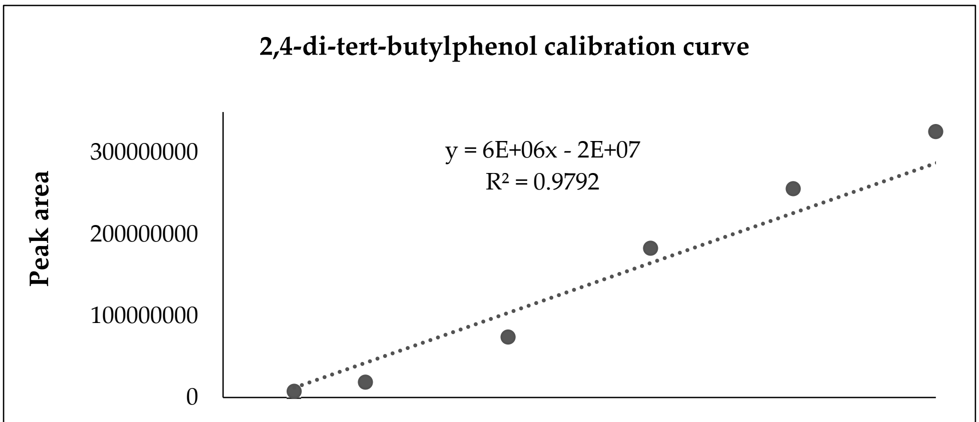

Figure S4. The calibration curve of 2,4-di-tert-butylphenol indicating the peak areas versus concentrations.

Table S4. Concentrations of 2,4-di-tert-butylphenol determined in extracts. DCM, EA, and MeOH are dichloromethane, ethyl acetate, and methanol, respectively. U.l: *U. lactuca* green alga. S.s: *S. scoparium* brown alga. P.c: *P. capillacea* red alga.

Nature of extract	Retention time (min)	Peak Area	Concentration (µg/mL)
**U.l (DCM)**	18.81	1764918.00	3.63
**U.l (EA)**	19.94	896260.01	3.48
**U.l (MeOH)**	19.94	304380.02	3.39
**S.s (DCM)**	19.93	159387.92	3.36
**S.s (EA)**	19.94	4878035.40	4.14
**S.s (MeOH)**	19.93	75100.34	3.35
**P.c (DCM)**	19.96	7226178.90	4.53
**P.c (EA)**	19.94	283623.88	3.38
**P.c (MeOH)**	19.95	470829.35	3.41

S5. Evaluation of the antibiofilm activity of 2,4-di-tert-butylphenol against *S. aureus*

The potential antibiofilm activity of the phenolic compound 2,4-di-tert-butylphenol (tested concentrations: 50.00, 25.00, 12.50, and 6.25 µg/mL, prepared in SDW) was evaluated using the protocol detailed in the Materials and Methods section (4.3). The obtained results are presented in Figure S5.

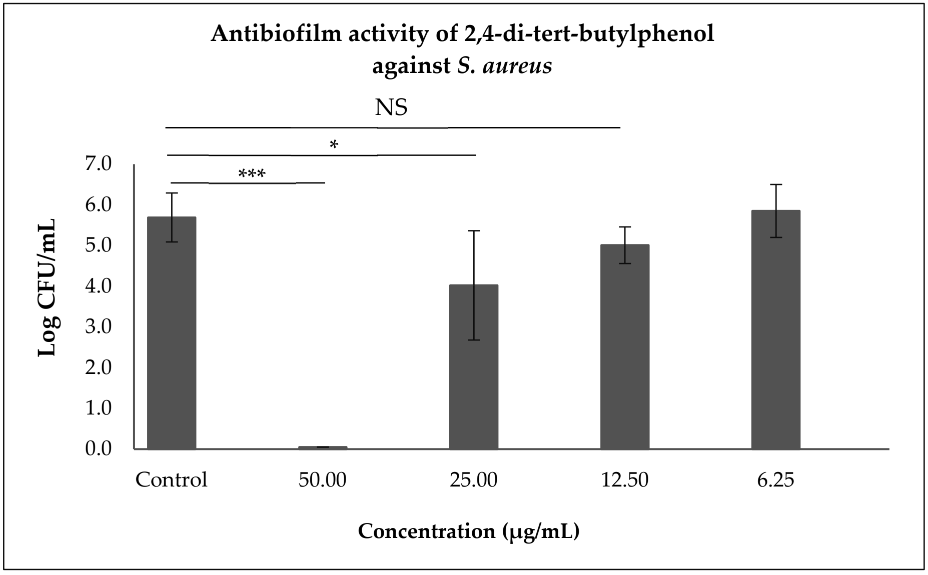

Figure S5. Effect of 2,4-di-tert-butylphenol (50.00, 25.00, 12.50, and 6.25 µg/mL) on *S. aureus* biofilm formation and growth. Product was added at t_0. Results are expressed as means of log CFU/mL ± SD from three independent experiments. Statistically significant differences (***, *p*-value < 0.001, and *, *p*-value < 0.05) between log CFU/mL number in the product treated biofilm and that in the untreated control are indicated. NS: not significant.
